# Resilience to COVID-19: Socioeconomic Disadvantage Associated With Higher Positive Parent-youth Communication and Youth Disease-prevention Behavior

**DOI:** 10.21203/rs.3.rs-444161/v1

**Published:** 2021-04-23

**Authors:** Andrew Marshall, Daniel Hackman, Fiona Baker, Florence Breslin, Sandra Brown, Anthony Dick, Marybel Gonzalez, Mathieu Guillaume, Orsolya Kiss, Krista Lisdahl, Connor McCabe, William Pelham, Chandni Sheth, Susan Tapert, Amandine Van Rinsveld, Natasha Wade, Elizabeth Sowell

**Affiliations:** Children's Hospital of Los Angeles; University of Southern California; SRI International; Laureate Institute for Brain Research; University of California, San Diego; Florida International University; University of California, San Diego; Stanford University; SRI International; University of Wisconsin–Milwaukee; University of California, San Diego; University of California, San Diego; University of Utah; University of California, San Diego; Stanford University; University of California, San Diego; Children's Hospital of Los Angeles

**Keywords:** Socioeconomic disadvantage, Resilience to COVID-19, positive parent-youth, youth disease-prevention, behavior

## Abstract

Socioeconomic disadvantage is associated with larger COVID-19 disease burdens and pandemic-related economic impacts. We utilized the longitudinal Adolescent Brain Cognitive Development Study to understand how family- and neighborhood-level socioeconomic disadvantage relate to disease burden, family communication, and preventative responses to the pandemic in over 6,000 youth-parent/caregiver dyads. Data were collected at three timepoints (May to August 2020). Here, we show that both family- and neighborhood-level disadvantage were associated with parents’ reports of greater family COVID-19 exposure risk and diagnoses, less perceived exposure risk, more frequent parent-youth conversations about COVID-19 risk/prevention and reassurance, and greater youth preventative behaviors. More disadvantaged families may be adaptively incorporating more protective strategies to reduce emotional distress and likelihood of COVID-19 infection. The results highlight the importance of parent-youth communication and disease-preventative practices for buffering the economic and disease burdens of COVID-19, along with policies and programs that reduce these burdens for families with socioeconomic disadvantage.

## Introduction

Like the 1918 Spanish and 2009 H1N1 influenzas,^1^ the SARS-CoV-2 (COVID-19) pandemic has impacted lower-income populations more heavily than higher-income populations.^2,3^ There is greater COVID-19 infection risk, prevalence, and disease severity in lower-income and more disadvantaged regions (i.e., neighborhoods, counties),^4-13^ and state-level income inequality is associated with COVID-19-related deaths.^14^ By the spring and summer of 2020, there was evidence that neighborhood disadvantage was associated with greater COVID-19 prevalence in several regions across the United States.^15,16^ Some research also suggested that greater risks of COVID-19 infection and death are linked with lower family-level household income.^17,18^ All the while, those from more disadvantaged families and neighborhoods are more likely to suffer unemployment or other economic shocks,^19^ as nearly 50% of lower-income American adults have either taken a pay cut or lost their job due to the COVID-19 pandemic, compared to 32% of higher-income individuals.^20^ Thus, families and communities at higher risks of suffering the health and economic consequences of the pandemic ultimately have fewer resources to manage disease outbreaks, thereby worsening and exacerbating extant economic and health disparities.^1^

These intersecting economic and health burdens highlight the importance of how children and families emotionally and behaviorally respond to manage challenging circumstances and reduce risk. One example concerns the preventive actions taken to reduce COVID-19 infection risk. Sociodemographic and community risk may be associated with barriers to following public health guidelines, such as differences in job-related risk or transportation,^21^ even though many such behaviors (e.g., social distancing, handwashing, mask-wearing) effectively mitigate infection risk and remain central components of public health messaging and pandemic response strategies.^22-24^ However, parent-child interactions and their relationships may be important factors influencing how youth manage the risk and prevention of COVID-19 infection and their pandemic-related worries and concern.^25^ Recent theoretical models have highlighted the role of parent-child communication in such resilience processes (i.e., sensitive and transparent communication about COVID-19 and youth’s emotional states),^26,27^ and some early data suggest that parent-child communication about COVID-19 may be protective for mental health, although measurement of communication in earlier reports was limited and not informed by consideration of family and community disadvantage.^28^ Ultimately, parent-child communication may be both a potential risk-reduction process and buffer to help children stay safe and foster resilience in the face of COVID-19-related adversities.

The Adolescent Brain Cognitive Development^SM^ Study (ABCD Study®; i.e., a large, national cohort of 11,878 youth and parent participants across 21 metropolitan areas in the United States, hereafter “ABCD”) uniquely allows for investigating how family-level (from parent and youth self-reports) and geocoded neighborhood-level predictors are associated with the unequal “costs” of the pandemic on American families.^1^ Past studies reporting associations between neighborhood disadvantage and COVID-19 have employed ecological analyses of associations between community characteristics and community- or census-tract level COVID-19 prevalence (or other small-area levels). Accordingly, these studies are limited in their abilities to focus on both family-specific disease burden and adaptive responses to the pandemic,^8- 13,15,16^ and capture the independent roles of family and neighborhood-disadvantage, which, while correlated, have separable associations with health that may operate by different mechanisms and, thus, warrant different intervention approaches.^29– 33^

In contrast, ABCD allows for the integration of neighborhood-level characteristics with family and youth data through census-tract-level geocoding of participants’ primary residence at baseline data collection (between 2016–2018). The geocoded data include the area deprivation index (ADI), a 17-variable composite metric of neighborhood disadvantage derived from the U.S. Census Bureau’s American Community Survey (ACS). ABCD has independently collected three waves of COVID-19-related questionnaire data from its youth participants and their caregivers/parents from May to August 2020, including inquiries about disease burden, perceived risk, parent-child communications, pandemic-related worry, and preventative behaviors to reduce the spread of the virus.

The goal of this study is to characterize associations between neighborhood disadvantage, family (household) income, and the disease burden of COVID-19 disease risk and worry, along with family-level interactions that may alter COVID-19 perceptions, help youth manage their worries, and foster preventative actions to reduce their risk of infection with COVID-19. We hypothesized that lower household income and greater neighborhood disadvantage would be associated with greater disease burden (i.e., family risk and exposure to COVID-19), greater perceived risk of exposure/infection, and greater COVID-19-related worry. We also hypothesized that any discrepancies in the predicted relationships between socioeconomic disadvantage and COVID-19-related risk and worry may be due to differential factors in the immediate family environment related to fostering resilience and encouraging preventative actions. To our knowledge, this is the first account of how family- and geocoded neighborhood-level disadvantage are associated with parent and youth responses to the pandemic within a large sociodemographically diverse cohort from a geographically heterogenous sample of US communities.

## Results

### Demographics and Analysis

Analyses included 16,017 observations across 6,874 unique participants (Table 1). Incomplete youth-parent/caregiver dyad data (e.g., parents/caregivers, hereafter referred to as “parents”, but not their children returning Q1 or vice versa) was not an exclusionary criterion. Relative to the entire ABCD cohort, our surveyed sample was more likely to (1) have higher incomes, (2) live in less disadvantaged census tracts, (3) identify the youth’s race as white, and (4) identify the youth’s ethnicity as non-Hispanic (Table 1). Here, ADI covaried with household income, Spearman’s rho (ρ) = 0.50, *p* < .001. At Q1, youth participants were ~ 12.5 years old (range: 10.6–14.6).

Primary analyses employed linear mixed-effects models with linear and quadratic terms of household income and ADI as the predictors of interest, controlling for race, ethnicity, parental education, and youth age and sex at birth for analyses of youth data or parent data about the youth participant (see Tables S4-S35). ABCD study site and participant ID were included as random effects. Family risk/exposure analyses incorporated generalized linear mixed-effects models. Statistical significance was assessed by comparing each coefficient divided by its standard error to the *t*-distribution.

### COVID-19 Disease Burden: Family Exposure Risk and Reported Diagnoses

**Figure 1:**
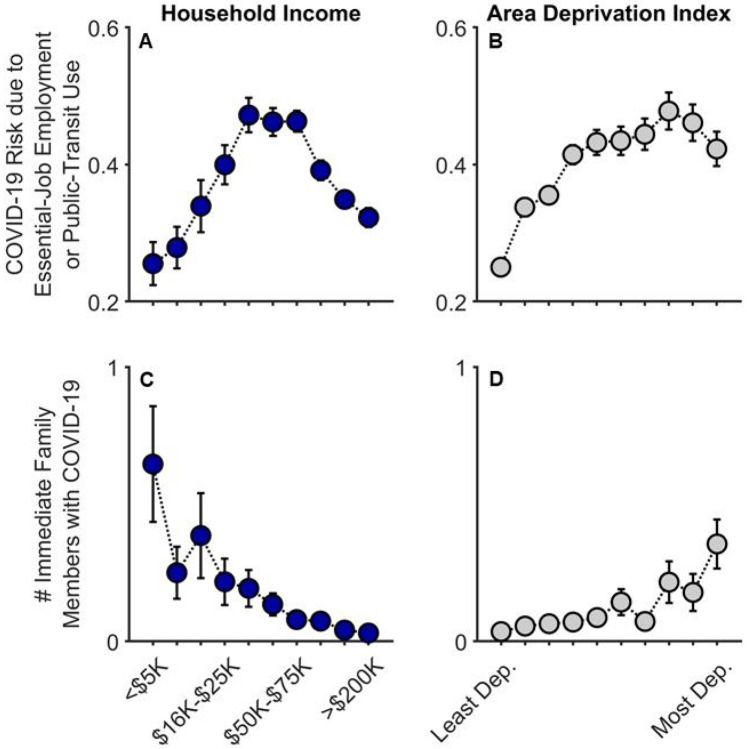
COVID-19 exposure and within-family diagnoses as functions of annual household income and their home census tract’s area deprivation index. Parent-reported data are shown for whether individuals in participants’ households were at an increased risk given job type or public-transit use (A, B) and the number of participants’ immediate family members who had been diagnosed with COVID-19 (C, D). Error bars represent ±1 between-subjects standard error of the means. Analyses controlled for caregiver/parental education, caregiver/parent race, caregiver/parent ethnicity, and participants’ baseline study site. Given multiple observations, the job/transit risk analysis also controlled for questionnaire number and participant. Area deprivation index was collapsed across continuous deciles for graphing. Dep. = Deprived.

Across Q1-Q3, risk of COVID-19 exposure due to essential-job employment or public-transit use significantly increased with ADI (i.e., greater neighborhood disadvantage), *t*(15313) = 10.45, *p* < .001, partial correlation coefficient (*r_p_*) = .084, but this pattern plateaued across the highest ADI tracts [(ADI)^2^], *t*(15313) = 5.48, *p* < .001 (Figs. 1A-B; Table S4). While risk of exposure generally increased with household income, *t*(15313) = 9.14, *p* < .001, *r_p_* = .074, there was a substantial decrease across the largest household incomes [(household income)^2^], *t*(15313) = 6.07, *p* < .001, in that, for household income, intermediate income households were those more likely at increased risk of exposure.

At Q2 (late June to July 2020), 3.4% of parents (178/5223) reported that at least one immediate family member (i.e., same household) had been diagnosed with COVID-19. Families with lower household incomes, *t*(5210) = −5.52, *p* < .001, *r_p_* = .076, and those living in higher ADI census tracts, *t*(5210) = 3.74, *p* < .001, *r_p_* = .052, reported more family members having been diagnosed with COVID-19 (Figs. 1C-D; Table S5; also see Table S3 for a detailed breakdown of these data). In the highest ADI decile (most disadvantaged neighborhoods), 10.1% of families reported having at least one family member diagnosed with COVID-19; in the most affluent neighborhoods, 2.7%. Similarly, while 12.5% of the lowest-income households reported that at least one family member had been diagnosed with COVID-19, 2.5% of the highest-income households reported at least one COVID-19 diagnosis; for families with the lowest household incomes who also lived in both the most disadvantaged neighborhoods, 17.6%. Thus, as predicted here and consistent with previous reports,^15-17^ lower household incomes and residence in greater ADI census tracts were associated with greater familial COVID-19 disease burden and, thus, greater risk of diagnosed COVID-19 exposure for parents and youth.

### Perceived Risk

Contrary to our predictions, decreased household income was associated with decreases in believing that the participant his-/her-/themself would get COVID-19, *t*(10081) = 5.42, *p* < .001, *r_p_* = .054, that someone close to them would get COVID-19, *t*(10082) = 7.10, *p* < .001, *r_p_* = .071, and that someone close to them would be hospitalized or die from COVID-19, *t*(10082) = 2.96, *p* = .003, *r_p_* = .030 (Tables S6-S9).

Associations of ADI with perceived risk were considerably weaker than with household income (Tables S6-S9). Nonetheless, similar to findings with household income, greater neighborhood disadvantage (higher ADI) was associated with significant decreases in participants believing he/she/they would get COVID-19, *t*(10081) = 2.57, *p* = .010, *r_p_* = .026, and that someone close to them would get COVID-19, *t*(10082) = 2.07, *p* = .038, *r_p_* = .021; the other perceived-risk relationships with ADI were not significant, *p*s ≥ .491.

As higher ADI and lower income were associated with having one or more family members diagnosed with COVID-19, we conducted sensitivity analyses including only those who had *not* had immediate family members diagnosed with COVID-19 to examine the possibility that perceived risk may differ based on experiencing positive COVID-19 tests within the household. The relationships with household income were maintained (albeit weaker) for thinking that one’s self, *t*(7992) = 4.22, *p* < .001, *r_p_* = .047, or someone close to him/her/them would get COVID-19, *t*(7993) = 5.59, *p* < .001, *r_p_* = .062, and for whether someone close to him/her/them would be hospitalized and/or die from COVID-19, *t*(7993) = 2.10, *p* = .036, *r_p_* = .023 (Table S10-S13). However, upon accounting for reported rates of diagnosis, ADI was no longer associated with perceived risk of exposure, *p*s ≥ .092, suggesting that perceived risk may not align with actual disease burden or likelihood of infection.

### COVID-Related Worry

Lower household income was related to greater parental worry, *t*(15357) = 2.87, *p* = .004, *r_p_* = .023. Parental worry also tended to increase with ADI in higher ADI tracts [(ADI)^2^], *t*(15357) = 2.05, *p* = .041] (Fig. 3A-B; Table S14). Although youth worry decreased with increasing household incomes, *t*(12510) = 2.32, *p* = .020, *r_p_* = .021, with these levels plateauing at greater income levels [(household income)^2^], *t*(12510) = 2.90, *p* = .004, youth worry was neither linearly nor quadratically related to ADI, *p*s ≥ .072 (Table S15). Parent-reported youth worry levels about the health- and non-health-related consequences (e.g., financial) of COVID-19 were also negatively associated with household income, *p*s < .001, but not ADI, *p*s ≥ .342 (Tables S16-S17). Analyses also indicated that greater disease burden was related to increases in parent but not youth worry levels (see Supplemental Information, “COVID-19-Related Worry and Disease Burden”).

Youths’ worry levels were highly correlated with, but noticeably lower than, their parents’ worry levels, Spearman’s rho (ρ) = .28, *p* < .001 (Fig. 3C) (Parent: *M* = 3.01, *SEM* = 0.01; Youth: *M* = 2.36, *SEM* = 0.01). While youth were only asked about general COVID-19-related worry, youth’s self-reported worry was more highly correlated with their parents’ report on their health-related, ρ = .26, *p* < .001, than non-health-related worry, ρ = .14, *p* < .001 (Fig. 3D), suggesting that youth’s general COVID-19-related worry was more related to their concerns about getting sick from COVID-19 rather than its non-health-related consequences.

### Families’ Responses to the COVID-19 Pandemic

Family-level disadvantage (i.e., lower household income) was associated with both increased disease burden (risk/exposure) and increased youth and parent worry, while neighborhood-level disadvantage (i.e., higher ADI) was associated with increased disease burden. However, increased socioeconomic disadvantage across both levels was associated with reduced perceived risk. To determine whether more disadvantaged families were differentially engaging in potential coping or disease-risk reduction strategies given increased COVID-19 risk and disease burden, we analyzed indicators of parent-youth communication about COVID-19 risk and prevention, parental reassurance about COVID-19, parental transparency with their child regarding their own COVID-19-related concerns, and youth’s COVID-19 preventative behaviors.

#### Parent-Youth Communication about COVID-19 Risk and Prevention

Lower household income was associated with increased communication on all topics queried regarding COVID-19 prevention (Fig. 4A): the importance of handwashing, *t*(15063) = 3.12, *p* = .002, *r_p_* = .025; the importance of social distancing, *t*(15062) = 5.03, *p* < .001, *r_p_* = .041; cancellations of school and other events, *t*(15061) = 5.27, *p* < .001, *r_p_* = .043; avoiding visits with friends/family, *t*(15061) = 7.11, *p* < .001, *r_p_* = .058; COVID-19 symptoms, *t*(15057) = 6.08, *p* < .001, *r_p_* = .049; protecting the elderly/vulnerable, *t*(15061) = 4.55, *p* < .001, *r_p_* = .037; and, wearing masks, *t*(4804) = 3.34, *p* = .001, *r_p_* = .048 (Tables S22-S28). In other words, families with lower incomes were speaking with their children about COVID-19 prevention more frequently than their higher-income counterparts were. Aside from parents’ talking about COVID-19 symptoms and handwashing, *p*s ≥ .359, these associations tended to plateau at the highest income levels [(household income)^2^], *p*s ≤ .028.

For ADI, while there were small negative associations between ADI and frequency of parent-youth discussions on three of the queried COVID-19 prevention topics [importance of social distancing, *t*(15062) = 2.81, *p* = .005, *r_p_* = .023; avoiding visits with friends/family, *t*(15061) = 3.06, *p* = .002, *r_p_* = .025; and, wearing masks, *t*(4804) = 2.34, *p* = .019, *r_p_* = .034], there were significant positive quadratic terms for ADI for each of these topics, *p*s ≤ .016 (Fig. 4B; Tables S22-S28). To better understand the quadratic relationships between ADI and parent-youth communication on COVID-19 prevention, we conducted bivariate ADI-by-parent/youth-communication correlational probe analyses (for all prevention topics) separately for those with ADI ≤ 40th percentile (Low ADI; *n* = 4,414 participants) and for those with ADI > 40th percentile (High ADI; *n* = 2,460 participants), given the minimal change in parent-youth communication below the 40th percentile (i.e., the 40% least deprived per national percentile; Fig. 4B). For High ADI participants, there were significant positive correlations between ADI and parent-youth communication frequency on all queried topics related to COVID-19 risk/prevention, ρs ≥ .12, *p*s < .001. In contrast, these relationships were substantially weaker for Low ADI participants (hand washing: ρ = .01, *p* = .484; social distancing: ρ = .04, *p* < .001; cancellations: ρ = .01, *p* = .338; avoiding visits: ρ = .04, *p* < .001; COVID-19 symptoms: ρ = .02, *p* = .041; protecting the elderly/vulnerable: ρ = .01, *p* = .252; wearing masks: ρ = .04, *p* = .018), further suggesting that parents in more disadvantaged neighborhoods were talking more with their children about COVID-19 prevention (Fig. 4).

Parents were also asked about how often they engaged in parental reassurance (i.e., “everything will be okay”) and parental encouragement to not dwell on COVID-19, as well as items related to transparent parental communication (i.e., parents’ discussing their own feelings about COVID-19, avoiding discussions about COVID-19, and discussing with their child about the safety and life-altering impact of COVID-19).

Lower household income was associated with more parental encouragement, *t*(9839) = 2.72, *p* = .007, *r_p_* = .027, but there was no relationship between income and parental reassurance, *t*(9842) = 1.18, *p* = .239, *r_p_* = .012 (Tables S29-S30). Except for talking about their own COVID-19-related feelings, *p* = .698, parents with lower household incomes were more likely to avoid talking to their child about COVID-19, *t*(9842) = 5.09, *p* < .001, *r_p_* = .051, more likely to tell their child that they may not be fully safe from COVID-19, *t*(9841) = 4.40, *p* < .001, *r_p_* = .044, and more likely to prepare their child that their lives may change significantly, *t*(9842) = 4.28, *p* < .001, *r_p_* = .043 (Fig. 5A; Tables S31-S34). Higher ADI (greater neighborhood disadvantage) was associated with more parental reassurance, *t*(9842) = 2.58, *p* = .010, *r_p_* = .026, and encouragement, *t*(9839) = 2.43, *p* = .015, *r_p_* = .024 (Fig. 5B; Tables S29-S30). However, there were no linear or quadratic associations with ADI and parental transparency items, *p*s ≥ .300 (Fig. 5B; Tables S31-S34).

Therefore, like parent-youth communication on COVID-19 risk/prevention, parents with lower household incomes and/or living in higher ADI tracts may be providing their child with greater preventative and anxiety-reducing emotional support in the wake of increased risk of COVID-19 exposure. Increased frequency of parent-youth discussions on COVID-19 prevention was also associated with less perceived risk, particularly in High ADI participants (see Supplemental Information, “Parent-Youth Communication and Perceived Risk”).

#### Youths’ Preventative Actions

Given increased disease burden (i.e., exposure, risk) and more frequent parent-youth discussions on COVID-19 prevention in disadvantaged families, we analyzed how often these children endorsed engaging in COVID-19-reducing behaviors (i.e., average frequency across multiple items: wearing a mask, avoiding others inside and outside their house, using hand sanitizer, washing hands, wiping surfaces, and avoiding touching people and things).

In the face of increased COVID-19 disease burden within families, youth of lower-income families and those living in more disadvantaged neighborhoods reported greater engagement in preventative actions. Greater household income was associated with decreased frequency of youths’ preventative actions, *t*(8071) = 4.05, *p* < .001, *r_p_* = .045, plateauing at the greatest household incomes [(household income)^2^], *t*(8071) = 3.48, *p* = .001 (Fig. 6A, Table S35). An increase in youth preventative actions was also evident in the more disadvantaged neighborhoods [(ADI)^2^], *t*(8071) = 3.41, *p* = .001 (Fig. 6B). As with parent-youth communication frequency, there was a strong positive relationship between ADI and youth preventative actions for High ADI participants, ρ = .16, *p* < .001, but a weaker, negative relationship for Low ADI participants, ρ = .07, *p* < .001.

The direct relationships between socioeconomic disadvantage and youths’ engagement in preventative behaviors mirrored the relationships with how often parents reported discussing prevention with their children. This finding was confirmed via a strong association between the average frequencies of youths’ preventative actions and parent-youth discussions on COVID-19 risk and prevention, ρ = .30, *p* < .001 (Fig. 6C). While youth who were more worried about COVID-19 also engaged more in COVID-19 preventative actions, ρ = .28, *p* < .001 (Fig. 6D), parents who were more worried about COVID-19 were also more likely to talk to their children about COVID-19 risk and prevention strategies (see Fig. 3), ρ = .37, *p* < .001 (Fig. 6E). More frequent parent-youth discussions on COVID-19 prevention and greater youth engagement in preventative behaviors were also associated with increased parental support and transparency (see Supplemental Information, “Preventative Actions, Parental Support, and Parental Transparency”). Thus, frequency of parent-youth communication on COVID-19 risk/prevention paralleled how often children endorsed engaging in preventative actions, both occurring more often in families with lower household incomes and/or those living in more disadvantaged census tracts.

## Discussion

**Figure 2:**
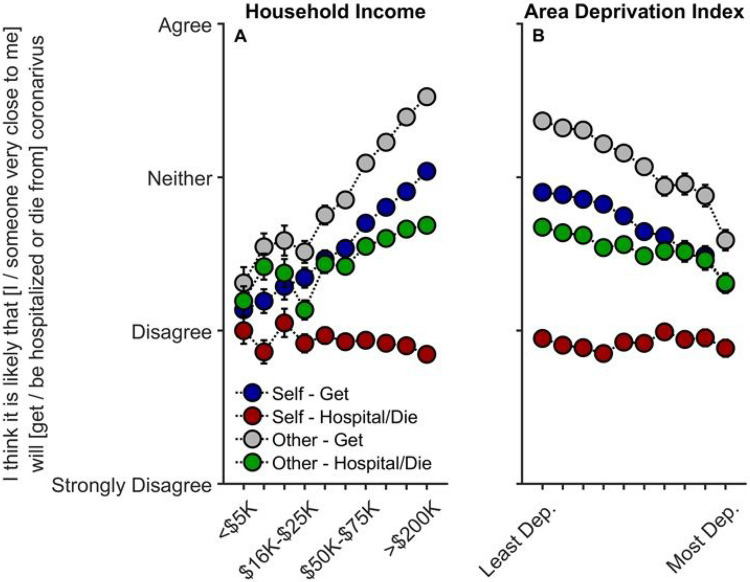
Parents’ perceived risk of their (or someone close to them) getting and being hospitalized/dying from COVID-19 as functions of (A) annual household income and (B) their home census tract’s area deprivation index. With respect to questionnaire item wording, “Self” refers to “I”, and “Other” refers to “someone very close to me”. Error bars represent ±1 between-subjects standard error of the means. Analyses controlled for caregiver/parental education, caregiver/parent race, caregiver/parent ethnicity, questionnaire number, participants’ baseline study site, and participant. Area deprivation index was collapsed across continuous deciles for graphing. Dep. = Deprived.

**Figure 3:**
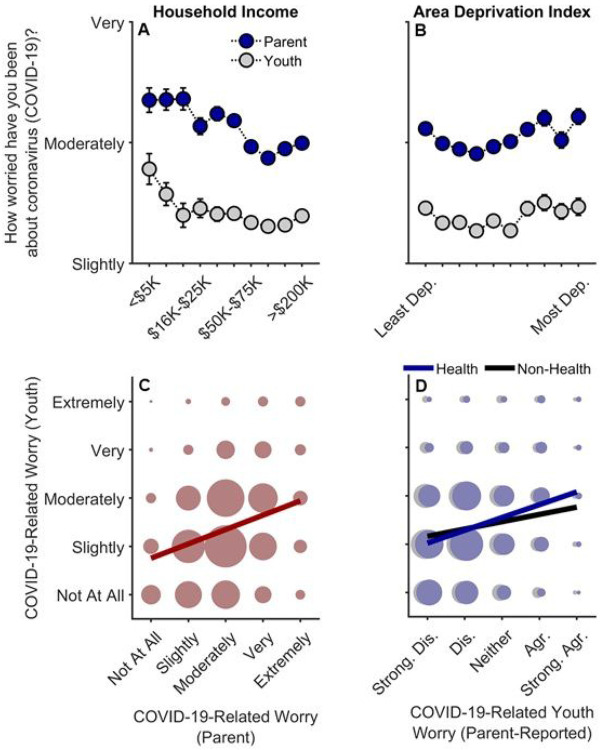
Parental and youth worry levels about COVID-19. (A, B) Worry levels as functions of annual household income and area deprivation index. Error bars represent ±1 between-subjects standard error of the means. Analyses of parental worry controlled for caregiver/parental education, caregiver/parent race, caregiver/parent ethnicity, questionnaire number, participants’ baseline study site, and participant ID. Analyses of youth worry controlled for caregiver/parental education, child race, child ethnicity, child sex, child age, questionnaire number, participants’ baseline study site, and participant. Area deprivation index was collapsed across continuous deciles for graphing. (C) Youths’ worry levels by parents’ worry levels. (D) Youths’ worry levels by parent-reported youth worry levels about the health- and non-health-related consequences of the COVID-19 pandemic. (C, D) The size of the circles reflects the number of datapoints at each x-y coordinate. The solid lines are best fitting simple regression lines. Dep. = Deprived. Strong. Dis. = Strongly Disagree. Dis. = Disagree. Agr. = Agree. Strong. Agr. = Strongly Agree.

**Figure 4:**
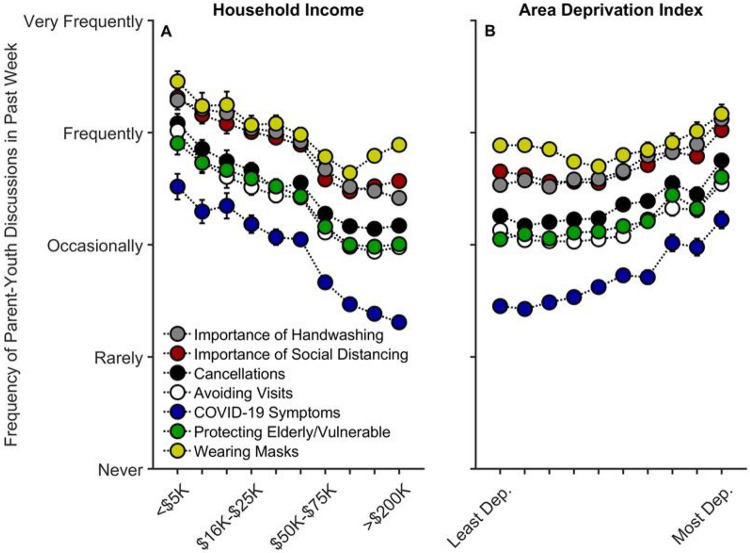
Parent participants’ communication frequency with youth on factors related to COVID-19 risk and prevention as functions of annual household income and their home census tract’s area deprivation index. Error bars represent ±1 between-subjects standard error of the means. Analyses controlled for caregiver/parental education, caregiver/parent race, caregiver/parent ethnicity, child sex, child age, questionnaire number, participants’ baseline study site, and participant, except for “Wearing Masks”, the analysis for which did not include questionnaire number or participant ID due to its only having one timepoint. Area deprivation index was collapsed across continuous deciles for graphing. Dep. = Deprived.

**Figure 5:**
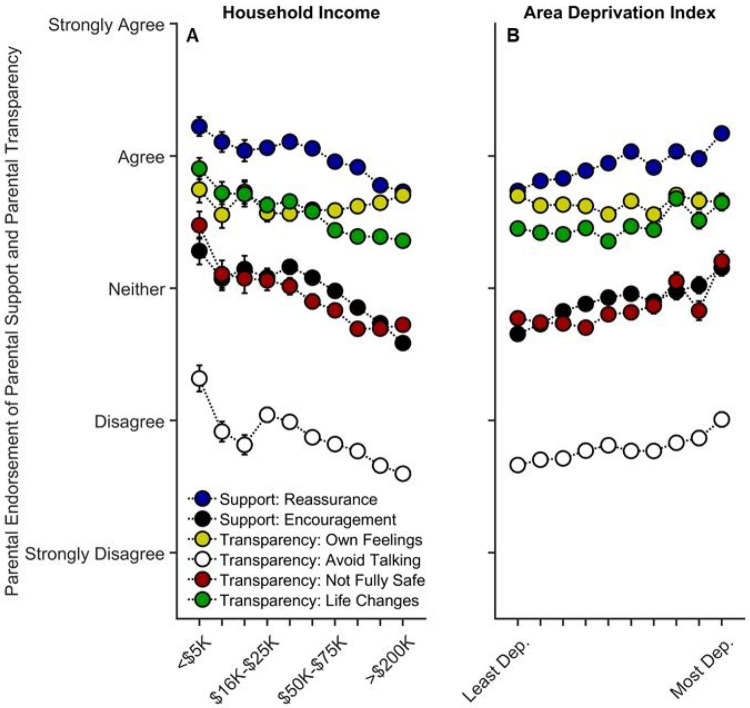
Parental support and transparency as functions of annual household income and their home census tract’s area deprivation index. Error bars represent ±1 between-subjects standard error of the means. Analyses controlled for caregiver/parental education, caregiver/parent race, caregiver/parent ethnicity, child sex, child age, questionnaire number, participants’ baseline study site, and participant. Parental “reassurance” refers to how much parents agreed with, “I have told my child that everything will be okay.” Parental encouragement refers to how much parents agreed with, “I have encouraged my child not to focus on coronavirus or its impacts on people and the world.” “Own Feelings” refers to how much parents agreed with “I discussed with my child my own feelings about coronavirus and its impact on people and the world.” “Avoid Talking” refers to how much parents agreed with, “I have avoided talking to my child about coronavirus.” “Not Fully Safe” refers to how much parents agreed with, “I have expressed concern to my child that they might not be fully safe from coronavirus.” “Life Changes” refers to how much parents agree with, “I have prepared my child for our lives to change significantly.” Area deprivation index was collapsed across continuous deciles for graphing. Dep. = Deprived.

**Figure 6:**
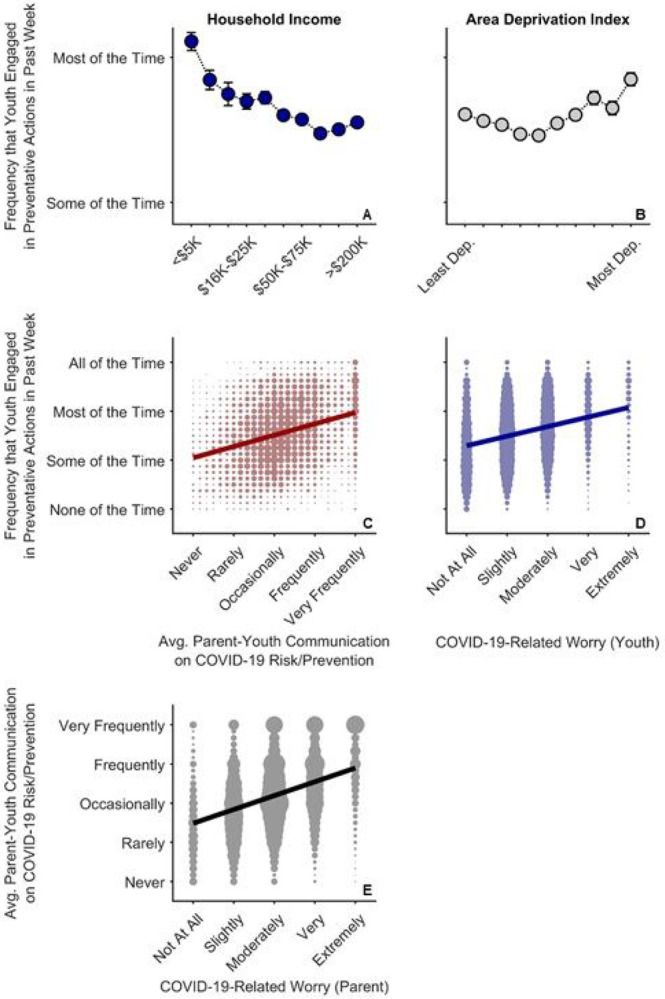
COVID-19 risk and prevention as functions of COVID-19-related worry in parents and youth. (A,B) Average (Avg.) frequency that youth endorsed engaging in COVID-19-related preventative behaviors as functions of annual household income and area deprivation index. Error bars represent ±1 between-subjects standard error of the means. Analysis controlled for caregiver/parental education, child race, child ethnicity, child sex, child age, questionnaire number, participants’ baseline study site, and participant. Area deprivation index was collapsed across continuous deciles for graphing. (C) Frequency of youths’ preventative behaviors by parent-youth communication frequency on risk/prevention, which are the averaged data from Figure 4. (D) Frequency of youths’ preventative behaviors by youth COVID-19-related worry. (E) Frequency of parent-child communication on risk/prevention by parent COVID-19-related worry. (C-E) The size of the circles reflects the number of datapoints at each x-y coordinate. The solid lines are best fitting simple regression lines.

To our knowledge, this is the first study describing associations between how parents and their children are responding to COVID-19, with respect to disease burden, perceived risk, communication, emotional distress, and behaviors to reduce its spread, in the context of family- and neighborhood-level socioeconomic disadvantage. As in previous reports,^1-18^ we showed greater COVID-19 disease burden in households with lower incomes and/or living in more disadvantaged neighborhoods (Fig. 1). However, in contrast to research employing ecological analyses of the socioeconomic disparities of COVID-19’s impact, our report uniquely integrated both neighborhood- and family-level socioeconomic data to elucidate how multilevel socioeconomic disadvantage in a nationwide sample related to parents’ and youths’ responses to the ongoing pandemic. While worry levels were higher among families with family-level socioeconomic disadvantage (Fig. 3A), families with greater family- and neighborhood-level disadvantage reported more parent-youth discussion on ways to reduce the spread of COVID-19 (Fig. 4), more frequent supportive and transparent discussions about COVID-19 between parents and their children (Fig. 5), and more frequent youth preventative actions (Fig. 6). Protective actions in these more disadvantaged families may have contributed to less perceived risk of COVID-19 infection (i.e., less belief of getting or being hospitalized/dying from COVID-19; Fig. 2; also see Figure S1). Ultimately, separable associations with neighborhood- and family-level socioeconomic disadvantage suggest that consideration of the disproportionate impact of COVID-19, family responses, interventions, or policy approaches to reduce the corresponding inequities must consider both families and their communities.^32^

Past reports have described how family- and neighborhood-level factors may heighten vulnerabilities to natural/manmade disasters and disease outbreaks,^1-3^ such as the 9/11 terrorist attacks^34^ and Hurricane Sandy.^35^ The COVID-19 pandemic has been no exception. While those of higher socioeconomic status (SES) may have been exposed to COVID-19 earlier in the pandemic,^7^ potentially experiencing the greatest changes to their daily lives,^36^ the pandemic has disproportionately burdened the families with lower household incomes and/or those living in more disadvantaged regions.^2,8-13,15-17,37^ The Centers for Disease Control’s Social Vulnerability Index (SVI) (https://www.atsdr.cdc.gov/placeandhealth/svi/index.html) and related SVI metrics have also been linked to higher COVID-19 case and death rates.^4-6,8^ While our analyses revealed similar patterns (i.e., increased family risk and exposure given greater socioeconomic disadvantage), our data provide unique insight into how these differential levels of disadvantage are associated with individual parent-youth processes and behaviors given such ecological risks. Overall, our results suggest that more disadvantaged parents and families may be proactively taking steps to reduce disease burden, suggesting that the necessary public health and policy interventions to reduce inequitable burdens of COVID-19, and, perhaps, reduce mental health problems that emerge from the pandemic, would be strengthened by collaborating and coordinating with communities, building on their strengths to focus on prevention.

Previous research has shown that adolescents (13–18 years old) with stronger views on the severity of COVID-19 were more likely to engage in social distancing and disinfecting behavior.^38^ Along with research showing that greater COVID-19-related worry^39^ and fear^40^ were related to more behavioral change in adults, our results demonstrate that COVID-19-related worry was highly correlated with parental and youth engagement in behaviors related to risk-reduction and prevention (Fig. 6). As disease burden was more closely aligned with COVID-19-related concern and preventative action, the reduction in perceived risk given lower household incomes and higher ADI (Fig. 2) may be partially due to heightened vigilance related to the pandemic (i.e., participants may be less likely to think that they or someone close to them will get COVID-19 because they are taking more preventative action to reduce its spread; Figure S1). Parents may be acting as buffers for how their children are emotionally and behaviorally responding to COVID-19, in that youths’ COVID-19-related worry and response may better reflect their parents’ worries than the state of the surrounding community (i.e., how neighborhood disadvantage is associated with COVID-19 disease burden). While children of lower-income families may be more cognizant of their own families’ SES, they may be less affected by community risk if their parents adaptively incorporate strategies to reduce environmental influences of COVID-19 infection and any associated emotional distress.

During the pandemic, public transit use in Chicago and New York City declined less in more disadvantaged areas, which are home to many “essential” workers,^41,42^ suggesting that those living in regions most vulnerable to COVID-19^8,9,15,16^ may not have the same luxury to engage in the same COVID-19 prevention efforts as more advantaged individuals.^7,21,42,43^ Papageorge, et al. ^43^ showed that although higher-income individuals were more likely to engage in COVID-19-related protective behaviors, those who had experienced losses to household income did so as well. While we cannot infer causality, our data suggest that disadvantaged individuals may be partially counteracting such elevated vulnerability via frequent discussions with their children on COVID-19 risk and prevention actions, even despite potentially greater costs to engage in COVID-19-preventative behaviors.^43^ As the relationship between parental stress and parental involvement in their children’s emotional regulation may be more pronounced in socioeconomically at-risk (than non-at-risk) families, with parental involvement being potentially more effective at reducing children’s negative emotions in at-risk families,^36^ it is imperative to develop strategies to support disadvantaged families during (and in the aftermath of) the global crisis brought upon by the pandemic.

Our analyses revealed many statistically significant quadratic terms, most apparent in the increased-risk disease-burden analyses for household income (Fig. 1). Here, households with relatively intermediate household incomes expressed the greatest COVID-19 risk given job type and public transit use, a pattern possibly been driven by both occupation type and employment (e.g., greater likelihood of working remotely in higher-income households). In Nigerian residents and healthcare workers, depressive symptoms were most prevalent in the intermediate income group.^44^ While our report does not focus on general mental health, polytonic relationships between family- and neighborhood-level SES and COVID-19-related risk, behavior, and prevention should be examined to identify unique risk factors for policy intervention.

Our results offer critical insights into associations between family- and neighborhood-level disadvantage and how parents and their children are responding to the COVID-19 pandemic, but these are not without limitations. The observational nature of ABCD precludes inferring causality regarding ADI and household income, as well as the directionality of parent-youth dyadic behavior (e.g., whether COVID-19-related discussions were parent- or child-initiated). However, an emerging strength of ABCD is its longitudinal design in a large cohort, permitting, for example, continued analyses of youth development with respect to differential exposures to COVID-19. Also, while the current report uses self-reports of disease burden, preventative behaviors, etc., the established rapport with ABCD families across study sites and similar patterns for multiple phenomena across parent and youth reports provide confidence in the data. Given ABCD’s rigorous biospecimen collection protocol (e.g., saliva, blood), ABCD is exploring methodology to incorporate COVID-19 tests and antibody testing in future protocols. This will be an advantage over self-report data, as data for total family members diagnosed with COVID-19 may be underestimations of true case rates, especially for the socioeconomically disadvantaged families who may have limited access to testing locations and vaccinations.^45^

With respect to youth preventative actions, we cannot distinguish between those who did not leave their homes (and, e.g., did not need to wear a mask) versus those who did (and, e.g., chose not to wear one). ABCD is currently geocoding several metrics related to social distancing/mobility (via SafeGraph; https://www.safegraph.com/), COVID-19 prevalence, unemployment, and local policy (e.g., closures, reopenings) to elucidate socioeconomic patterns of COVID-19-related preventative behavior and impact. Lastly, the ADI used here was based on participants’ primary residential addresses at baseline data collection of ABCD, a metric based on the 2011–2015 five-year ACS summary. Even though the ADI data are based on data from years before the onset of the pandemic, research has shown that deprivation levels of individuals’ neighborhoods are often relatively stable over time, even when participants move,^46^ a phenomenon that may persist across generations,^47^ suggesting that geocoding of addresses collected at baseline may be a sufficient proxy for participants who have moved.^48^ While nearly half of participants in the current sample were of higher-income families, our sample still encompassed the demographic diversity of the ABCD cohort with respect to including individuals living in the most disadvantaged neighborhoods with the lowest household incomes (see Table 1). It remains possible that this sample is not representative of the population, particularly for more disadvantaged families, further highlighting the need to prioritize research on and provide support for the disadvantaged families who will carry the heaviest burdens of the COVID-19 pandemic.^1-3^

In conclusion, our data suggest that more disadvantaged families may be promoting greater resilience in their children (or protecting them from greater COVID-19 disease burden) per more frequent discussions on COVID-19 risk/prevention, greater parental support, and more direct COVID-19-related conversations. Youth in more disadvantaged situations also reported greater preventative behaviors to reduce the likelihood of contracting COVID-19. While self-report COVID-19 data will continue to be invaluable to better understand how disasters impact adolescent development, contextualizing these data with respect to neighborhood factors^32^ may greatly inform how community leaders, policy makers, healthcare workers, and caregivers can alleviate the economic, health, and psychological impact of such disasters. Our results have critical implications for COVID-19-related physical and emotional health of children and their parents whilst educators and government officials consider the many factors to reopen schools and businesses to full capacity.^49^ In addition to the much needed actions to reduce disparities that contribute to disease risk, it may be helpful to encourage parental guidance as well as open COVID-19-related discussions between parents and their children on preventative behaviors, which may reduce socioeconomic inequalities of COVID-19 disease burden, promote resilience to natural disaster in children, and encourage individuals to modify their own behaviors to proactively mitigate the scourge of the next pandemic. As ABCD progresses, its linking of residential history and other geocoded data to the COVID-19 questionnaire data will provide key insight into how early and current environments are associated with the health and mental health outcomes related to COVID-19 as well as subsequent trajectories of brain, emotional, social, and cognitive development.^50^ We urge public officials to aid and support disadvantaged families beyond the actions that they are already incorporating themselves, so as to mitigate, and eventually eliminate, the persistent unequal socioeconomic and health burdens that are unveiled and exacerbated in times of crisis.

## Methods

### Participants

ABCD is a 10-year longitudinal study involving 21 U.S. study sites.^51^ Using school-based enrollment,^52^ ABCD enrolled 11,878 9- and 10-year-old children from an initial 22 sites. The recruitment process and the derivation of the demographically diverse target sample has been previously described.^52^

In May 2020, ABCD began disseminating questionnaires to all parent and youth participants to assess how the COVID-19 was impacting their lives. Data from the first three questionnaires (disseminated by email on May 16–22, 2020, June 24–27, 2020, and August 4–5, 2020 via unique links from ABCD) are available through the National Institute of Mental Health Data Archive (NDA),^53^ which includes data for 9,268 total participants, with 5,125 youth-parent dyads both completing Questionnaire (Q) 1; 5,189, Q2; and 5,011, Q3. A total of 3,286 youth-parent dyads completed Q1 and Q2, and 2,915 participants completed Q1-3.

Our analyses incorporated main-study data from the November 2020 ABCD 3.0 data release (baseline data for 11,878 participants, 1-year-follow-up data for 11,235 participants, and 2-year-follow-up data for 6,571 participants).^54^ Centralized IRB approval was obtained from the University of California, San Diego. Study sites obtained approval from their local IRBs. For the main study, parents provided written informed consent; children provided written assent. Accessing the COVID-19 questionnaires (i.e., clicking on the secure link) indicated willingness to participate. Data collection and analysis complied with all ethical regulations.

### COVID-19 Questionnaire

Youth and parent participants were emailed the COVID-19 Questionnaire; a $5 incentive was provided for completing each questionnaire (~ 10–15 min to complete). The analyzed items from the questionnaires are shown in the Supplemental Materials (Table S2), with some items asked only at select timepoints. Youth questionnaires were provided in English, and parent questionnaires were available in English and Spanish. Data were collected via REDCap.^55,56^

#### COVID-19 disease burden: Family exposure risk and reported diagnoses.

COVID-19 disease burden was operationally defined as parents’ responses to two items capturing COVID-19-related burden and exposure: “Was anyone in your household at increased risk for COVID-19 due to work in healthcare or other essential jobs (such as grocery store, factory, gig economy) or use of public transit?” (Response options: No, Yes, Don’t Know; Q1-3) and “Number of immediate family members (same household) diagnosed with coronavirus” (0–10+; Q2).

#### Perceived risk of COVID-19.

Perceived risk was operationally defined as parents’ responses to four items (5-point Likert scale: Strongly Disagree, Disagree, Neither Disagree or Agree, Agree, Strongly Agree; Q1 and Q3): “I think it is likely that I will get coronavirus,” “I think it is likely I will be hospitalized or die from the coronavirus,” “I think it is likely that someone very close to me will get coronavirus,” and “I think it is likely that someone very close to me will be hospitalized or die from the coronavirus”.

#### Youth and parental worry.

In Q1-3, youth and parent participants rated how worried they had been about COVID-19 in the past week (5-point Likert scale: Not at All, Slightly, Moderately, Very, Extremely). Parent participants also reported on their children’s worry levels about the health and non-health related consequences of COVID-19 (5-point Likert scale: Strongly Disagree, Disagree, Neither Disagree or Agree, Agree, Strongly Agree; Q1 and Q3): “My child seems worried about becoming ill or that others they know will become ill with coronavirus,” and “My child seems worried about non-health related consequences of coronavirus (e.g., financial).”

#### Parent-youth communication about COVID-19 risk and prevention.

Parents were asked how often in the past week (5-point Likert scale: Never, Rarely, Occasionally, Frequently, Very Frequently; Q1-3) they talked with their child about (1) “the importance of handwashing for preventing the spread of germs,” (2) “the importance of social distancing,” (3) “cancellation of school and other events,” (4) “avoiding visiting friends or family,” (5) “the symptoms of coronavirus,” and (6) “protecting the elderly or other vulnerable people.” In Q3, “the importance of wearing a mask” was added. When specified, these responses (except for “wearing a mask,” as it was only asked at Q3) were averaged for analyses, for the purpose of simplicity.

#### Parental support and transparency.

Parental support was operationally defined as the extent to which parents agreed (or disagreed) with two items (5-point Likert scale: Strongly Disagree, Disagree, Neither Disagree or Agree, Agree, Strongly Agree; Q1 and Q3): “I have told my child that everything will be okay,” and “I have encouraged my child not to focus on coronavirus or its impacts on people and the world.” These items are referred to as *Parental Reassurance* and *Parental Encouragement,* respectively.

Parental transparency was operationally defined by parents’ agreement with four statements (5-point Likert scale: Strongly Disagree, Disagree, Neither Disagree or Agree, Agree, Strongly Agree; Q1 and Q3): “I discussed with my child my own feelings about coronavirus and its impact on people and the world,” “I have avoided talking to my child about coronavirus,” “I have expressed concern to my child that they might not be fully safe from coronavirus,” and “I have prepared my child for our lives to change significantly.”

#### Youths’ preventative actions.

Youth participants were asked about their frequency of engaging in preventative behaviors (4-point Likert scale: “I have not done this in the last week,” “I did this some of the time last week,” I did this most of the time last week,” “I did this all the time last week”; Q1 and Q3): (1) “I stay away from people (other than those who live in my house),” (2) “I wash my hands at times other than just after I use the bathroom or before eating,” (3) “I wear a mask over my face or protective gear (e.g., gloves, things to cover my clothes),” (4) “I use Purell/other hand sanitizer,” (5) “I use Clorox/cleaners to wipe down surfaces,” (6) “I avoid touching things (e.g., phone, doorknobs),” (7) “I avoid touching people (e.g., hugging, shaking hands),” and (8) “I stay away from people inside my house (e.g., stay in another room or a certain distance away).” For analysis, these Likert scale data were averaged across items.

### ABCD Main-Study Data

Analyses incorporated family demographics and residential history data collected as part of the primary ABCD Study (Table S1).

#### Socioeconomic Disadvantage

The area deprivation index (ADI) for youth participants’ primary residential address at the baseline visit is a composite weighted-sum metric of neighborhood disadvantage (e.g., poverty rates, unemployment, median family income, low education; see Table S1).^57,58^ Census-tract-level ADI, based on the 2011–2015 five-year ACS estimates, was computed based on coefficient values from Kind, et al. ^57^ and discretized into national percentiles for the ABCD data release. The code for computing and merging ADI (and its national percentile) with ABCD data is available: https://github.com/ABCD-STUDY/geocoding/blob/master/Gen_data_proc.R. While statistical analyses incorporated the national percentile ADI data, these data were collapsed across continuous deciles for graphing.

Parent-reported annual household income (before taxes, including all wages and benefits) was a continuous, ordinal factor with 10 levels (1 = <$5,000; 2 = $5,000-$11,999; 3 = $12,000-$15,999; 4 = $16,000-$24,999; 5 = $25,000-$34,999; 6 = $35,000-$49,999; 7 = $50,000-$74,999; 8 = $75,000-$99,999; 9 = $100,000-$199,999; 10 = ≥$200,000). For income, the data used in analyses were those of the most recent, non-missing data available for that participant for each of these options (i.e., these variables were collected at each annual visit but the multiyear recruitment period of ABCD means that those annual visits were staggered across participants).

#### Demographic Covariates

Youth participants’ ages and sex at birth were available in the COVID-19 data release. Children’s and caregiver’s race and ethnicity were categorical factors derived from parent reports at baseline data collection. Race had 6 levels: “White”, “Black”, “Asian”, “American Indian or Alaska Native”, “Native Hawaiian or Other Pacific Islander”, or “Other” (e.g., multiracial). Ethnicity had two levels: “Hispanic/Latino/Latina” or “Not Hispanic/Latino/Latina”. Maximum parental education was a continuous, ordinal factor with 5 levels (1 = ≤ 12th grade, no diploma; 2 = high-school graduate, GED or equivalent; 3 = Some college with no degree, Associate’s degree; 4 = bachelor’s degree; 5 = master’s degree, professional degree, or doctorate). As with the income data, the education data used in analyses were the most recent, non-missing education data available for that participant.

### Statistical Analyses

The integration of COVID-19 questionnaire data and ABCD main-study data resulted in 21,646 data points across 9,268 participants. Participants’ data were excluded listwise if the primary residential address was invalid (remaining *n* = 20,483), if the ADI score was missing or invalid (weighted sum = 0) (remaining *n* = 20,079), if there were missing data for household income, sex, age, parental education, race, ethnicity (remaining *n* = 19,012), or if any of a participant’s questionnaires were returned out of order (e.g., Q1 was completed after Q2 was completed) (remaining *n* = 18,731). Individual questionnaire data were excluded for a participant if that questionnaire was returned after the dissemination date of the subsequent questionnaire (see above) (remaining *n* = 18,476). (Q3 responses were excluded if they were returned after October 8, 2020, the dissemination date of the fourth COVID-19 questionnaire). While ABCD includes siblings, issues of convergence of random-effects structures led us to include only one sibling per multiparticipant family. If siblings had completed a different number of COVID-19 questionnaires, then the sibling with the most questionnaires completed were included. If multiple siblings had completed the same number of questionnaires, then the sibling included in analyses was randomly selected using MATLAB’s *datasample* function (seed = 1). Omnibus analyses included 16,017 data points (i.e., questionnaire responses) across 6,874 participants. (Note that “participants” here refers to at least one member of parent-child dyad, as some parents but not their children returned the questionnaires at each timepoint, and vice versa.)

Analyses employed MATLAB’s Statistics and Machine Learning Toolbox 11.7 (R2020a; MathWorks). Model output and model-fit characteristics are provided in the Supplemental Materials (Tables S4-S35). Statistical reporting in the main text is in the form of t-statistics. Effect sizes of main associations for continuous factors are represented by partial correlation coefficients (*r*_p_), which control for all model covariates and are calculated using the corresponding t-statistic and degrees of freedom^59^; for uniformity, effect sizes were also calculated using this approach for generalized linear mixed-effects models. Likert-type response data were analyzed with general linear mixed-effects models (with a random initial value for iterative optimization). Count data were analyzed with generalized linear mixed-effects models assuming a Poisson distribution and a log link function. Questionnaire data with yes-no responses (excluding “Don’t Know” responses) were analyzed with generalized linear mixed-effects models assuming a binomial distribution and a logit link function (0 = no, 1 = yes). For each analysis, missing data (or missing-like data, e.g., “Don’t Know” responses) were excluded on a pairwise basis. Categorical factors were effects-coded to facilitate interpretation of main effects; continuous factors were centered to make parameter estimates more interpretable.^60^ Bivariate Spearman correlational analyses were also conducted when specified.

Mixed-effects analyses incorporated fixed effects of ADI, maximum parental education, questionnaire number (centered for analysis), household income, race, and ethnicity. Analyses of data from parent-completed questionnaires included parent race and ethnicity; those from youth-completed questionnaires, youth race and ethnicity. Analyses of parent-reported youth worry levels included youth race and ethnicity. The fixed-effects structure of analyses of youth questionnaire data (or parent questionnaire data directly about their child, e.g., support, transparency, communication, worry) also included youth sex and age. After having conducted preliminary analyses of these data, quadratic terms for maximum parental education, household income, ADI, and questionnaire number were also included as fixed effects. (Questionnaire number was not included as a fixed effect in analyses with only a single time point, and a quadratic term for questionnaire number was not included when there were only two time points.)

Random-effects structures included random intercepts for participant ID (for analyses of questionnaire items with repeated observations) and study site; here, because ADI referred to that of participants’ residences at baseline, the study site for each participant was also that from baseline data collection (i.e., some participants in the ABCD Study have been transferred from their baseline site to other sites over the first 4 years as a function of family relocation).

## Supplementary Material

Supplement 1

## Figures and Tables

**Table 1:** Demographics for the Adolescent Brain Cognitive Development (ABCD) Study

	Release 3.0 (%) [Baseline Data]	Sample in this Report (%)
**Youth Sex**		
Male	6,196 (52.1%)	3,612 (52.5%)
Female	5,682 (47.8%)	3,262 (47.5%)
**Annual Household Income**		
<$5,000	417 (3.5%)	168 (2.4%)
$5,000-$11,999	421 (3.5%)	187 (2.7%)
$12,000-$15,999	274 (2.3%)	137 (2.0%)
$16,000-$24,999	524 (4.4%)	254 (3.7%)
$25,000-$34,999	654 (5.5%)	342 (5.0%)
$35,000-$49,999	934 (7.9%)	503 (7.3%)
$50,000-$74,999	1,499 (12.6%)	926 (13.5%)
$75,000-$99,999	1,572 (13.2%)	983 (14.3%)
$100,000-$199,999	3,315 (27.9%)	2,371 (34.5%)
≥$200,000	1,250 (10.5%)	1,003 (14.6%)
Missing/Undefined	1,018 (8.6%)	0 (0.0%)
**Area Deprivation Index**		
≤ 33 Percentile (Low)	5,392 (45.4%)	3,655 (53.2%)
34–66 Percentile (Mid)	3,499 (29.5%)	2,162 (31.5%)
≥ 67 Percentile (High)	2,055 (17.3%)	1,057 (15.4%)
Missing/Undefined	932 (7.8%)	0 (0.0%)
**Youth Race**		
American Indian / Alaska Native	62 (0.5%)	26 (0.4%)
Asian	276 (2.3%)	192 (2.8%)
Black	1,869 (15.7%)	822 (12.0%)
Native Hawaiian / Pacific Islander	16 (0.1%)	8 (0.1%)
Other	1,959 (16.5%)	1,096 (15.9%)
White	7,525 (61.1%)	4,730 (68.8%)
Missing/Undefined	171 (1.4%)	0 (0.0%)
**Youth Ethnicity**		
Hispanic	2,411 (20.3%)	1,258 (18.3%)
Not Hispanic	9,314 (78.4%)	5,616 (81.7%)
Missing/Undefined	153 (1.3%)	0 (0.0%)
**Total**	11,878 (100%)	6,874 (100%)
